# The severity of facial burns, dental caries, periodontal disease, and oral hygiene impact oral health-related quality of life of burns victims in Pakistan: a cross-sectional study

**DOI:** 10.1186/s12903-021-01923-3

**Published:** 2021-11-08

**Authors:** Farooq Ahmad Chaudhary, Basaruddin Ahmad, Mohd Zulkarnain Sinor

**Affiliations:** 1 School of Dentistry, Shaheed Zulfiqar Ali Bhutto Medical University, Islamabad, Pakistan; 2 School of Dental Sciences, Universiti Sains Malaysia, Kubang Kerian, Malaysia

**Keywords:** Oral health status, Oral health-related quality of life, Oral hygiene, Quality of life, Facial burns, Risk factors, Patient-based outcomes

## Abstract

**Background:**

A burn to the face and neck area leaves a visible scar that impacts the victims physically and psychologically. This report was aimed to examine the factors associated with oral health-related quality of life (OHRQoL) in patients with a facial burn injury.

**Methods:**

Patients with facial burn who attended the Burn Care Centre in Islamabad, Pakistan were systematically and randomly invited to participate in this cross-sectional study. They underwent extra- and intra-oral examinations and, completed self-administered instruments in the Urdu language. The severity of disfigurement, dental caries experience (DMFT), periodontal disease (CPI) and oral hygiene (OHI-S) statuses were assessed. The validated instruments collected information relating to sociodemographic background, oral health behaviours, OHRQoL and satisfaction with appearance (SWAP). Information relating to the time of the incident, cause and severity (type, TBSA) of the burn were obtained from medical records. The OHRQoL prevalence of impact and severity measures were derived and analysed using simple and multiple, logistic and linear regression.

**Results:**

A total of 271 patients had participated in the study. The OHIP-14 prevalence of impact was 94% with mean severity score = 37 unit (sd = 8.5). The most impacted domains were physical pain (87%), psychological disability (87%), social disability (85%) and physical discomfort (83%). The main determinants of oral health-related quality of life were poor clinical oral conditions - particularly caries, and severity deformity. Other risk factors included poor oral health behaviours, psychological distress and longer time elapsed since the incident, and sex (*p* < 0.05).

**Conclusion:**

Dental caries, the severity of the facial deformity, oral health behaviour and time are associated with oral health-related quality of life of patients with facial burns. Oral health behaviour improvement can lower the risk of developing dental problems and oral health-related quality of life impact.

## Background

Burn injuries have an immediate and long-term impact on the victims, family members and healthcare services; it limits and/or disables functions and physical health, disfigures appearance, causes pain, and affect the social and psychological well-being of survivors for the rest of their lives [1–4]. The major causes of injury are direct contact with heat, radiation, electricity, friction and chemicals, relating to occupation, accident and assault [5–9]. Flames and acid burn are common injuries to the head and neck caused by home accidents and assault [10, 11].

A burn to the face and neck area leaves a visible scar that impacts the victims physically and psychologically. Changes such as tissue loss, scarring, altered motor structural morphology, loss of elasticity, and limited mobility and function occur immediately at the affected area following a burn [12, 13]. As the scar tissue matures over time, it contracts and further deforms the appearance, leading to incompetent lips, limitation of mouth opening, incomplete oral occlusion and, for joint contracture, temporomandibular joint dysfunction and restriction of movement; these affect daily oral functions such as eating, swallowing and speaking. In children, the force and tension of the scar contracture can affect the growth of the maxilla and mandible that later cause excessive anterior teeth protrusion and crowding [14, 15]. Stretching of scar tissues during mouth opening and jaw movement can cause discomfort and pain [12, 16–18]. These complications limit access to the oral cavity and disrupt oral hygiene care; the resulting inefficiency increases the risk of developing dental caries and periodontal diseases that later affect the oral health-related quality of life [1, 2, 19–29]. A study has shown that facial burn victims have poor oral conditions [30].

Studies have shown that facial disfigurement causes psychological distress in patients with a facial burn injury [2, 31] and that the psychological conditions are linked to poor dental conditions and oral health-related quality of life in the general population [32, 33]; however, there is limited discussion in the literature whether the severity of a facial burn injury is related to the oral health-related quality of life of the victims. The objective of this study was to examine the factors associated with oral health-related quality of life in patients with facial burns using two outcome measures derived from the Oral Health Impact Profile (OHIP-14) instrument.

## Methods

A cross-sectional study was carried out at the Burn Care Centre of the Institute of Medical Sciences, Islamabad, Pakistan. The study protocol was approved by the ethics committee of the institute (Reference no. F.1-1/2015/ERB/SZABMU) and written informed consent was obtained from all the participants and parents/guardians of participants under 15 years of age before data collection. Patients aged 15 years or older who were visiting the centre for follow-up treatment were systematically and randomly selected into the study if they presented with a burn injury that affected more than 10% of the total body surface area (TBSA), including the head and neck area and able to feed by mouth. Patients with a severe psychological problem and a burn-related hand injury that can affect proper manual oral hygiene maintenance were excluded. The sample size calculation showed that 270 patients were needed to meet the objectives based on the prevalence of periodontal disease, caries and the quality of life scores of each domain in OHIP-14 and the total score [34–36].

The participants underwent a facial examination and oral screening and completed a set of self-administered questionnaires. The severity of the facial deformity was assessed using the observer-rated disfigurement scale, which is a measure based on the size and degree of disfiguration, the extent of impairment on facial expression and visibility of the affected area, which ranges from 1 to 9. A greater rating indicates more severe disfigurement [37, 38]. The oral screening was carried out by one of the researchers (FAC) who is a qualified dentist. Caries experience was measured using the DMFT index according to the WHO survey method [39]. Periodontal disease and oral hygiene status were measured using the Oral Hygiene Index-Simplified (OHI-S) and Community Periodontal Index (CPI), respectively based on the methods described in earlier reports [40, 41]. An additional oral health measure, the *clinical oral status*, was derived by combining the DMFT, CPI and OHI-S indices using the Principal Component Analysis with no rotation which resulted in one latent measure that explained 82.8% of the variance with an Eigenvalue = 2.5 and mean = 0.0 (sd = 1.0; min =  − 3.17, max = 1.91) [42]. A higher value corresponds to a worse overall clinical oral condition.

The sociodemographic information collected were gender, age group, education, employment, income. The oral health behaviours included daily tooth brushing frequency (once/twice or more), frequent dental visits in the past year (Yes/No) and barriers to utilisation of oral healthcare services. The latter was adapted from Patton et al. by asking the participants whether factors such as cost, anxiety, location, illness, or other problems had prevented them from seeking oral health care services using an open-ended question. The first response was categorised as dental anxiety, social, distance, cost and self-perceived [43]. Oral health-related quality of life was assessed using the Oral Health Impact Profile (OHIP-14), a 14-item instrument that assesses the impact of oral health problems on seven domains: functional limitation, physical pain, psychological discomfort, physical disability, psychological disability, and social disability and handicap, in the past 12 months [35, 36]. Each item was scored as 0 (never), 1 (seldom), 2 (sometimes), 3 (often) or 4 (very often). Two outcome measures were derived. The severity score assesses the severity of impact and is calculated by adding the score of the 14 responses. It ranges from 0 to 56 units and an increasing value corresponds to a greater impact. The *prevalence* of impact is the percentage of participants who have at least one item impacted (score ≥ 3) [44, 45]. The Satisfaction With Appearance Scale-Urdu (SWAP-U) measures the perceived satisfaction with facial image and socio-behavioural impact of burn scars. It has 14-items in four domains: dissatisfaction with facial features, dissatisfaction with body appearance, social discomfort and perceived social impact, with the response ranging from strongly disagree (1) to strongly agree (7). The total score ranges from 0 to 84 units with a greater value indicating greater dissatisfaction [46, 47]. Information regarding burn injury was obtained from the medical records. Four measures were recorded: the classification of severity based on the depth of burn (second vs third-degree), total body surface area affected (10–20% and > 20%), time since the incident and cause of the burn.

### Statistical analysis

The summarise statistics of the sample and parameters were obtained using the descriptive, Pearson and Kendall’s tau correlation analyses. Based on the recommendation that reports on patient-based outcomes should include analysis of alternative scoring formats [48], analyses were carried out for both the OHIP-14 prevalence and severity score.

Analysis of the prevalence of impact was carried out using bivariate and multiple logistic regression. In cases with a total separation of observations whereby where all participants in a category are impacted, Firth logistic regression was used. Analysis of the severity score was performed using simple and multiple linear regression. The analyses assumed that the ordinal variables were linearly associated with the outcomes and treated each category in the categorical variables as an independent factor. Both multivariable analyses were performed using the hierarchical regression method. The analyses assumed that oral health status is the primary determinant of oral health-related quality of life, thus, the measures were included in the first block of independent variables as the fix factors. Next, the significant variables from the bivariate analysis (*p* < 0.05) were added to the model as covariates; the burn-related measures were added to the model in the second block and followed by the oral health behaviours and sociodemographic factors in the third block. Each covariate was added one at a time using manual iteration but following the forward selection technique and it is retained in the model if the R^2^ increased significantly with the probability of inclusion set at < 0.05 and removed if the *p*-value changes after more factors were added with the probability exclusion at > 0.1. Interaction terms between the sociodemographic factors were also examined.

The standardised coefficients of the final model from the multiple linear regression were contrasted to examine the strength of influence of a factor on oral health-related quality of life. Because of multicollinearity and overfitting issues in the multiple linear regression analysis [49], three alternative model solutions are presented. In Model 1, the DMFT, CPI and OHI-S were assumed to influence OHRQoL independently and included in the model as fixed factors. Models 2 and 3 assumed that they are related and hence, a composite measure, the clinical oral status, was used in the analysis as the fixed factor. Regression diagnostic analyses carried out on the models do not show a remarkable deviation from the assumptions. The significance level was set at 5% and analysis was done in the IBM SPSS v24.

## Results

A total of 300 patients with a facial burn injury were invited to participate in the study but 20 had declined to participate and 9 did not complete the questionnaire, hence, the total response rate with complete data was 90.3% (N = 271). The sample included 68.6% females, 78.9% below the age of 35-year-old, 91.9% with less than 12 years of schooling, 49.1% were unemployed and, 65.7% had low personal income (< 15,000 PKR or < US$140). Forty-eight per cent of the cases had a third-degree burn, 46.1% had more than 20% total body surface area (TBSA) burned and 82.7% had had the injuries for more than 2 years. The majority had fire-related burn injury (41.3%) and, between moderate to severe facial disfigurement (88.9%). The prevalence of caries (DMFT ≥ 1) was 100% with a mean DMFT = 11.0 (sd = 2.4), 59% had periodontal pockets > 4 mm and 66.1% had poor oral hygiene. Details of the sample background had been reported earlier [30]. The prevalence of OHRQoL impact was 94.1% (95%CI = 91.27%, 96.92%) and the mean severity score was 37.7 units (95%CI = 36.64, 38.67 units) (Table 1). The four most prevalent and severe domains impacted were physical pain (87%), psychological (87%) and social (85%) disabilities, and psychological discomfort (83%), which corresponded to the severity score.

**Table 1:** Summary of OHIP-14 add-score and impact prevalence by items and domains (N = 271)

Domain and item	Item severity score Mean (sd)	Domain severity score Mean (sd)	Item impact prevalence n %	Domain impact prevalence n%
*Functional limitation*				
1. Trouble pronouncing words	2.97 (.948)	5.21 (1.62)	201 (74.2)	204 (75.3)
2. Sense of taste worsens	2.25 (.864)		125 (47.2)	
*Physical pain*				
3. Has painful aching	2.92 (.587)	5.54 (1.20)	224 (86.7)	236 (87.1)
4. Uncomfortable to eat	2.62 (.797)		192 (71.9)	
*Psychological discomfort*				
5. Been self-conscious	2.72 (.893)	5.58 (1.71)	203 (75.0)	225 (83.0)
6. Feel tense	2.87 (.965)		202 (74.6)	
*Physical disability*				
7. Unsatisfactory diet	2.65 (.869)	5.04 (1.62)	166 (61.2)	179 (66.1)
8. Interrupt meals	2.39 (.895)		101 (37.2)	
*Psychological disability*				
9. Difficult to relax	2.97 (.867)	6.03 (1.88)	216 (79.8)	236 (87.1)
10. Embarrassment	3.06 (.983)		203 (74.9)	
*Social disability*				
11. Irritable with other people	3.25 (.956)	5.79 (1.88)	228 (84.1)	231 (85.2)
12. Difficulty doing usual jobs	2.54 (1.231)		141 (52.0)	
*Handicap*				
13. Less satisfying life	2.73 (.842)	4.47 (1.10)	176 (65.0)	185 (68.3)
14. Unable to function	1.74 (.831)		47 (17.3)	
*Prevalence*				
No impact		16.06 (3.26)		16 (5.9)
Impacted (1 + domain)		39.01 (6.71)		255 (94.1)
Severity score:		37.67 (8.50)		

The bivariate analysis showed that the severity and prevalence of impact were associated with the severity of injury and disfigurement, the longer time elapsed since the burn incident, cause of the burn, greater dissatisfaction with appearance, and poor dental conditions (*p* < 0.01) (Table 2). Oral health-related quality of life was poorer in male than in female participants.

**Table 2:** The crude odds ratio and regression coefficient for the bivariate association between the OHIP-14 prevalence and severity of impact and independent factors (N = 271)

	Prevalence of impact		Severity of impact	
	n (%)**	Crude OR (95%CI) *p*	Mean (sd)	Regression coefficient (95%CI) (*p*)
*Age*				
15–24	79 (88.8)	1.5	34.2 (10.48)	1.2 (− 0.12, 2.36)
25–34	123 (98.4)	(0.78–3.02)	40.7 (5.13)	(0.05)
35–44	37 (92.5)	0.2	36.2 (8.90)	
≥ 45	16 (94.1)		37.1 (8.72)	
*Gender*		0.3	39.6 (5.69)	− 2.9 (− 5.05, − 0.72)
Male*	83 (97.6)	(0.07–1.33)	36.7 (9.38)	(0.002)
Female	172 (92.5)	0.1		
*Education (years of schooling)*				
0–5*	69 (94.5)	1.2^4^	39.1 (7.43)	0.8 (− 2.64, 0.98)
6–12	164 (93.2)	(0.50–3.01)	36.7 (9.07)	(0.4)
≥ 13	22 (100)	0.7	39.5 (5.90)	
*Employment status*				
Full-time job*	46 (93.9)	1.0	36.6 (8.32)	− 0.2 (− 1.38, 1.06)
Part-time job	72 (94.7)	(0.58, 1.91)	39.4 (6.95)	0.8
Unemployed	124 (93.2)	0.9	37.1 (9.33)	
Others^1^	13 (100)		37.5 (7.76)	
*Personal income PKR*^*2*^				
5000–14,000*	168 (94.4)	0.8 (0.52–1.38)	37.4 (8.79)	− 0.3 (− 1.33, 0.80)
15,000–24,000	45 (95.7)	0.5	40.0 (6.41)	(0.6)
25,000–34,000	22 (91.7)		35.8 (9.33)	
≥ 3500	20 (90.9)		36.3 (7.76)	
*Family income PKR(US$)*				
15,000–24,000 (140- 230)*	133 (95.7)	0.7 (0.37–1.16)	37.8 (8.75)	− 0.5 (− 1.66, 0.73)
25,000–34,000 (240- 325)	56 (94.9)	0.1	38.4 (7.09)	(0.4)
≥ 35,000(≥ 335)	66 (90.4)		36.6 (9.06)	
*Severity of injury*		1		
2^nd^-degree burn*	125 (88.7)	34.3^4^ (2.56–4.39 × 10^3^)	33.2 (9.43)	9.1 (7.35, 10.8)
3^rd^-degree burn	130(100.0)	< 0.001	42.3 (3.39)	(< 0.001)
*TBSA*				
10–20%*	130 (89.0)	31.7^4^ (4.22–4.06 × 10^3^)	33.7 (9.49)	8.6 (6.79, 10.3)
> 20%	125 (100)	< 0.001	42.2 (3.48)	(< 0.001)
*Time elapsed*				
1–2 years*	31 (66.0)	79.4^4^ (11.2–1.01 × 10^4^)	22.5 (7.31)	6.8 (6.14, 7.53)
2–3 years	78 (100)	< 0.001	37.9 (4.52)	(< 0.001)
3–4 years	105 (100)		41.5 (3.08)	
≥ 4 years	41 (100)		44.2 (2.80)	
*Cause of burn*				
Electrical\others*	16 (66.7)	1	25.7 (11.10)	− 13.4 (− 16.75, − 9.96)
Scald\Steam	74 (96.1)	12.3 (2.94, 51.68)	37.9 (7.17)	− 1.2 (− 3.39, 1.08)
		0.001		0.3
Fire\Flame	108 (96.4)	13.5 (3.64, 50.04)	39.1 (7.20)	39.1 (37.68, 40.53)*
		< 0.001		< 0.001
Chemical\assault	57 (98.2)	28.5 (3.32, 245.05)	39.3 (7.42)	0.3 (− 2.17, 2.72)
		0.002		0.8
*Disfigurement*	0.34^3^ (< 0.001)	4.8 (2.54, 8.93) (< 0.001)	0.81^4^ (< 0.001)	4.6 (4.18, 4.96) (< 0.001)
*SWAP-U*	0.30^3^ (< 0.001)	1.3 (1.18, 1.43) (< 0.001)	0.75^4^(< 0.001)	0.8 (0.76, 0.74) (< 0.001)
*DMFT*	0.34^3^ (< 0.001)	3.7 (2.12, 6.52) < 0.001	0.87^4^ (< 0.001)	3.1 (2.87, 3.28) (< 0.001)
*CPI*				
Bleeding*	16 (51.6)	50.6^4^ (12.35, 4.7 × 10^2^)	19.6 (6.81)	7.4 (6.67, 8.14)
Calculus	79 (98.8)	< 0.001	35.6 (6.36)	(< 0.001)
4-5 mm pocket	117 (100)		41.4 (2.55)	
> 6 mm pocket	43 (100)		44.2 (2.59)	
*OHI-S*				
Good*	2 (28.6)	26.4^4^ (8.29, 1.35 × 10^2^)	17.9 (4.10)	11.5 (10.2, 12.8)
Fair	74 (87.1)	< 0.001	30.5 (9.36)	(< 0.001)
Poor	179 (100)		41.8 (3.21)	
*Clinical oral status*	0.33^3^ (< 0.001)	58.7 (7.9, 4.4 × 10^2^) (< 0.001)	0.87^5^ (< 0.001)	7.4 (6.88, 7.89) (< 0.001)
*Brushing frequency (daily)*				
Two or more*	47 (79.7)	13.3 (4.10, 42.99)	27.7 (9.81)	12.7 (10.77, 14.6)
Once	208 (98.1)	(< 0.001)	40.4 (5.54)	(< 0.001)
*Dental visit (past 12 months)*				
Regular*	33 (73.3)	17.2^4^ (5.92, 59.68)	28.2 (11.27)	8.7 (6.79, 10.62)
Episodic	205 (98.1)	(< 0.001)	39.3 (6.47)	< 0.001
Never	17 (100)		42.8 (3.30)	

^1^student or retired, ^2^Pakistani Rupees, ^3^Kendall’s tau, ^4^from Firth logistic regression, ^5^Pearson correlation coefficient, *reference category, ** For reference purposes only from Chaudhary et al., 2019[30]

The analyses did not find a model with more than one significant factor in the multiple logistic regression model and only the DMFT and clinical oral status were significant (*p* < 0.05) as presented in Table 2. In contrast, several model solutions with the comparable model fit (adjusted-R^2^ ≈ 0.8) were obtained from the multiple linear regression analysis. For brevity, three models are presented in Table 3 to demonstrate the consistency in the findings. Model 1 showed that the DMFT, CPI, oral health behaviours, cost, disfigurement, fire, scald, and sex-education interaction were significantly associated with OHRQoL and the caries measure had the largest standardised coefficient (β = 0.48). The significance of the OHI-S was lost in Model 1 likely due to collinearity with the other clinical indices. When each of the oral health measures was entered separately as the only fixed factor and all the covariates in Model 1 kept the same, the standardised coefficient of the DMFT (0.60, *p* < 0.001, adjusted-R^2^ = 0.81) remains the largest compared to the CPI (0.30, *p* < 0.001, 0.76) and OHI-S (0.22, *p* < 0.001, 0.75). The standardised coefficients of the latter two were smaller than the *disfigurement* in the respective models (0.45 and 0.52, *p* < 0.001).

**Table 3:** The result of multiple linear regression analysis examining the factors associated with oral health-related quality of life in facial burn patients (N = 271)

(n)	Model 1 (adjusted R^2^ = 0.81^f^)	Model 2 (adjusted R^2^ = 0.79^f^)	Model 3 (adjusted R^2^ = 0.79^f^)
	Regression coefficient (95%CI) (*p*) (standardised coefficient, β)		
Total	–	–	–
DMFT	1.7 (1.24, 2.17)	–	–
	(< 0.001)		
	(0.48)		
CPI	1.1 (0.22, 1.99)	–	–
	(0.01)		
	(0.12)		
OHI-S	1.0 (− 0.30, 2.38)	–	–
	(0.1)		
	(0.07)		
Clinical oral status	–	4.7 (3.64, 5.85)	4.7 (3.80, 5.64)
		(< 0.001)	(< 0.001)
		(0.56)	(0.56)
Brushing frequency	1.9 (0.45, 3.34)	2.0 (0.44, 3.50)	1.9 (0.41, 3.42)
	(0.01)	(0.01)	(0.01)
	(0.09)	(0.10)	(0.09)
Dental visit	1.6 (0.46, 2.65)	1.7 (0.57, 2.89)	1.5 (0.38, 2.67)
	(0.01)	(0.004)	(0.01)
	(0.09)	(0.1)	(0.08)
Cost as barriers to oral health care utilization	− 1.3 (− 2.36, − 0.24)	− 1.5 (− 2.63, − 0.37)	–
	(0.02)	(0.01)	
	(− 0.07)	(− 0.08)	
Time since burn event	–	1.1 (0.13, 2.05)	–
		(0.02)	
		(0.12)	
*Cause of burn*			
Scald	2.0 (0.84, 3.22)	1.4 (0.30, 2.47)	1.1 (0.08, 2.17)
	(0.001)	(0.01)	(0.04)
	(0.11)	(0.07)	(0.06)
Fire	1.1 (0.02, 2.21)	–	–
	(< 0.05)		
	(0.07)		
Disfigurement	0.9 (0.25, 1.48)	–	1.4 (0.85, 2.02)
	(0.006)		(< 0.001)
	(0.15)		(0.26)
SWAP	–	0.1 (0.14, 0.23)	–
		(0.03)	
		(0.11)	
Interaction: Sex-education	− 0.4 (− 0.76, − 0.05)	− 0.4 (− 0.76, − 0.01)	− 0.4 (− 0.77, − 0.03)
	(0.03)	(< 0.05)	(0.04)
	(− 0.06)	(− 0.06)	(− 0.06)

Model 2 showed that one unit increase in clinical oral status score was associated with a 4.7 unit increase in the severity score (*p* < 0.001) after adjusting for oral health behaviours, cost, disfigurement, satisfaction with appearance, the time passed since the burn incident, scalding injury, and sex-education interaction factors. Model 3 was an alternative solution to address the collinearity between the disfigurement and SWAP and, included the same covariates as Model 2 except for the cost and time. In the two latter models, the standardised coefficients of clinical oral status were consistently the largest (β = 0.56). Both facial deformities measures were significantly associated with OHRQoL; the disfigurement has the second-largest standardised coefficient in Model 3 (β = 0.26) and the SWAP, the third-largest in Model 2 (0.11).

## Discussion

The present study had examined the factors influencing the oral health-related quality of life in patients with a facial burn and found associations between the prevalence and severity of impact and poor oral status, the severity of the facial deformity, oral health behaviours, psychological distress, the time elapsed since the incident, and sex-education interaction. About 94% of the participants had at least one oral health-related quality of life domain impacted. The prevalence of impact is the highest ever reported based on the definition of impact used in this study; in contrast to 16%, 18%, 19%, and 31% for the general population of England and Australia [45], and dentate and edentulous people, respectively [33, 50]. Although a higher prevalence of impact at 91% has been reported previously using the same instrument, such as in the homeless people and patients with the temporomandibular joint disorder, the definition adopted in these studies is less commonly, whereby an impact includes the responses *occasional*, *often* and *very often* (score ≥ 2) [32, 51]; hence, not directly comparable with the current finding. The definition of impact in the present study is conventional and restrictive; hence, the high prevalence observed highlights the importance of the issue in dental public health [45]. Similarly, the severity of impact in the participants was also the highest ever reported (mean = 37.7 units) compared to patients with gastrointestinal and hepatic disorders, temporomandibular joint disorder, and socially vulnerable and underserved populations (mean severity score range: 8.8–27.8 unit) [32, 52, 53]. Nevertheless, comparison of impact between population-based on prevalence and scores should be done cautiously; differences can be attributed to characteristics of the sample, different methods of administering the instrument, bias due to non-participation in large studies and large floor effect caused by a large number of responses with low severity score in a large population study compared to an entirely handicapped sample such as in the present study [45].

The analysis revealed two main findings. First, because the standardised coefficients of the oral health measures, and caries, in particular, are consistently the largest in all the multivariate models compared to other factors, poor oral health condition is the major attribute of oral health-related quality of life in facial burn patients. This further supports the existing understanding of the relationship between poor oral health status and oral health-related quality of life [29]. In the present study, more than 70% of the participants have had a toothache and eating discomfort related to untreated carious teeth and, in more than 80%, dental problems make them feel self-conscious, tense, unable to relax, embarrass, irritable and uncomfortable during social interaction; these are much greater compared to less than 10% reported in earlier studies [32, 33, 45].

Second, participants with more severe facial deformities have a poorer oral health-related quality of life. Based on the four-dimensional OHRQoL model [54, 55], oral health conditions and facial deformity largely impact three of the four dimensions: oral function, orofacial pain, and psychosocial impact; the greatest being the psychosocial well-being of the participants in this study, which concur with the domains’ prevalence and severity of impact (Table 1). There is a lack of information from the present data to explain the relationship but there are plausible insights from reflections of the data collection process. Besides having poor oral health conditions, many of the participants also have disfigured lips, exposed and mobile teeth, dry mouth and halitosis, which make social interaction challenging to either party. They showed signs of discomfort, shyness, and distress particularly during the facial and oral examination [21, 38]. Some participants seemed upset for having to repeat themselves because the researcher did not understand their pronunciation. Experiencing these situations daily can affect self-esteem, and stress, anxiety, and depression levels. These are the likely explanation for the high frequency of *often* and *very often* OHIP responses that contributed to the high impact prevalence and severity score; a reporting behaviour that is in contrast with the denial attitude described in Slade et.al. (2005) as less willingness to report a severe impact [45]. Nevertheless, more evidence is needed to reaffirm the observations. A recent study showed that psychosocial distress can affect oral health through oral health behaviour [56].

The longer the time since the burn incident is associated with poorer OHRQoL and that is not unexpected because oral diseases continue to worsen without intervention [57]. The interaction between sex and education reflects the better OHRQoL in the females than males among the less educated participants, but it is worse among the better-educated females. More educated women are generally more expressive when describing a depressive impact because they are more conscious about their appearances and aesthetics than men [58, 59]. Income is not a significant factor because most participants in the study are from a less affluent background and there was little variation in the add-score between the different income levels. The high cost of treatment is a barrier to using oral health care services, but it has the least impact on oral health-related quality of life despite retaining its significance in the multiple regression, unlike psychological and self-perceived barriers. Similarly, electric burn also has the least impact on OHRQoL compared to other factors; possibly because it is less commonly associated with deep tissue damages to affect the oral environment [60]. The factor was replaced by scalding and fire burn in the multivariate analysis to demonstrate variations in model solutions and focus on more traumatising cause of burn. Nevertheless, it is important to note that gains and losses in the significance level observed in these measures are likely statistical artefacts resulting from variations in the measurement and the categorising process and does not reflect the real world. Therefore, the interpretation of their significance should be treated with great caution.

This study had analysed both outcome measures: the prevalence and severity of OHIP-14 impact. With exception of the sex, the factors associated with oral health-related quality of life are similar in the simple logistic and linear regression analyses. However, the total separation problem, a situation where there is zero observation (n = 0) in one of the cells, affects the precision of the odds ratio estimates, as observed in the confidence interval, and was the likely explanation for the poor fit of the multiple logistic models to the data and resulting in the non-significant covariates. Despite the recommendation [48], the data from this study contributed little to the currently limited discussion on the advantage of including more than one analysis of participant-based outcome measures and studies intending to adopt a similar approach should be aware of the data limitation. There are several equivalently fit models found in the multiple linear regression analysis as a result of multicollinearity problems. In Model 1, the correlation between the clinical indices causes the OHI-S to lose its significance. The use of clinical oral status in Models 2 and 3 resolved that issue and the results showing the effect of oral health problems on OHRQoL are consistent. Likewise, a similar problem between disfiguration and SWAP was resolved by examining them separately in Models 2 and 3. Except for the interaction mentioned earlier, other sociodemographic factors were excluded because they are not associated with oral health-related quality of life and do not significantly improve the models.

The results of this study should be interpreted with caution due to the limitations mentioned earlier and in related publications [30, 56]. A reliability study for the clinical measurements was not performed because the participants were not willing to return for re-assessment. Also, a limited application can be inferred to facial burn patients who are not followed up at a burn centre. The key strength of this study is its originality; this is the first report on OHRQoL of facial burn injury victims, a small niche and underserved population with unknown needs; it also showed that an acquired disability involving oro-facial area can affect oral health-related quality of life.

## Conclusion

Oral health problems, particularly dental caries, the severity of the facial deformity, oral health behaviour and time since the burn event are the determinants of oral health-related quality of life of facial burn victims. It is recommended that future studies investigate the oral health knowledge of, and problems relating to oral hygiene care faced by, patients with facial deformity; the understanding can be used to design interventions for the management of their specific needs. Facilitating access to oral health care and, early oral health education and counselling, and management of oral health needs can be beneficial to lower the impact. This is achievable by educating the burn-care professionals about the oral health needs of the victims and referring them to the dental office. Highlighting the issues of this population may help raise awareness about the oral health needs of the population among the relevant authorities.

## Data Availability

The datasets used and/or analysed during the current study are available from the corresponding author on reasonable request.

## Abbreviations

CPI: Community Periodontal Index
DMFT: Decayed, Missing, Filled Teeth
OHI-S: Simplified Oral Hygiene Index
TBSA: Total body surface area
WHO: World Health Organization
SWAP: Satisfaction with appearance scale
OHIP: Oral Health Impact Profile
